# Evaluation and correlation analysis of ocular surface disorders and quality of life in autoimmune rheumatic diseases: a cross-sectional study

**DOI:** 10.1186/s12886-023-02959-5

**Published:** 2023-05-22

**Authors:** Yuerong Ren, Jing Tian, Wen Shi, Jianing Feng, Yingyi Liu, Huanmin Kang, Baihua Chen, Yan He

**Affiliations:** 1 Department of Ophthalmology, The Second Xiangya Hospital, Central South University, Changsha, 410011 Hunan China; 2 Hunan Clinical Research Center of Ophthalmic Disease, Changsha, 410011 Hunan China; 3 Department of Rheumatology and Immunology, The Second Xiangya Hospital, Central South University, Changsha, 410011 Hunan China

**Keywords:** Dry eye disease, Autoimmune rheumatic diseases, Ocular surface, Quality of life, Anxiety and depression

## Abstract

**Objective:**

This cross-sectional study aimed to reveal the association between ocular surface disorders and psychological, physiological situations among autoimmune rheumatic patients.

**Methods:**

Ninety autoimmune rheumatic patients (180 eyes) hospitalized in the Department of Rheumatology, The Second Xiangya Hospital, Central South University and 30 controls (60 eyes) were enrolled in the study. All participants were assessed for ocular surface disorders including dry eye disease (DED) by the Ocular Surface Disease Index (OSDI) for symptoms evaluation, and slim lamp examinations for tear break-up time (TBUT), meibomian gland secretion, symblepharon and corneal clarity, Schirmer I test, corneal fluorescein staining (CFS), lid-parallel conjunctival folds (LIPCOF). Systematic conditions were evaluated using the Short Form 36-Health Survey (SF-36) for health-related quality of life, Hospital Anxiety and Depression Scale (HADS) for anxiety and depression, Health Assessment Questionnaire-Disability Index (HAQ-DI) for difficulties in activities of daily living, and Pittsburgh Sleep Quality Index (PSQI) for sleep quality. Pearson and spearman’s analysis were conducted to examine the relationship between systematic conditions and ocular surface conditions.

**Results:**

The analyses were controlled for age and sex. 52.22% of eyes (94 in 180) of autoimmune rheumatic patients and 21.67% of eyes (13 in 60) of controls were diagnosed with DED. The autoimmune rheumatic patients showed significant higher OSDI score, fewer basal tear secretion, more severe CFS and conjunctivochalasis than controls. There were no statistically significant differences in TBUT, meibomian gland secretion, symblepharon, and corneal clarity between the two groups. For systematic conditions, autoimmune rheumatic patients had significantly lower SF-36 scores, higher anxiety scores, and HAQ-DI scores than controls. No statistically significant differences were detected in depression scores and PSQI between the two groups. Among autoimmune rheumatic patients, OSDI scores were moderately correlated with quality of life, anxiety, depression and sleep quality.

**Conclusion:**

Factors including quality of life, anxiety, depression, and sleep quality are associated with ocular surface conditions, especially DED symptoms. Management of systemic conditions and psychotherapy should also be considered as part of the treatment among autoimmune rheumatic patients.

**Supplementary Information:**

The online version contains supplementary material available at 10.1186/s12886-023-02959-5.

## Key points

The autoimmune rheumatic patients have poor health-related quality of life and more severe ocular surface disorders compared to controls.

The subjective factors including quality of life, Anxiety, depression, and sleep quality are associated with ocular surface conditions, especially DED symptoms among autoimmune rheumatic patients.

## Introduction

Autoimmune rheumatic diseases are a series of chronic diseases that involve multiple systems, mainly including rheumatoid arthritis (RA), systemic lupus erythematosus (SLE), and Sjogren's syndrome (SS), etc. Patients with these diseases are often hospitalized due to severe arthritis, nephritis, or interstitial pneumonia, and pay less attention to ocular changes, especially in RA and SLE [1–3]. Studies have shown that ocular involvement is a sign of immune reactivation in many rheumatic diseases. In fact, ocular involvement is very common. A total of 90% of SS patients, 27% of RA patients and 31% of SLE patients had ocular involvement. Among them, ocular surface disorders are frequently reported, especially dry eye disease (DED), which is the most common ocular disorder [4]. Early eye examination and intervention are of great significance for the prognosis and outcome of these diseases [5, 6]. Additionally, in patients with SS, ocular examination assists in the diagnosis [7].

Ocular surface disorders may lead to blurred vision and decreased vision, further affecting work efficiency, severely reducing the quality of life and increasing the financial burden [8, 9]. In autoimmune rheumatic patients, the disease status and severity inevitably affect the degrees of stress, anxiety, and sleep quality[10, 11]. However, emotional disorders, poor sleep and some immunomodulatory medicine had been confirmed to deteriorate the ocular surface situations [12], especially intensified dry eye [13], which may impact the diagnosis and assessments of autoimmune rheumatic disease [11, 14]. It becomes a vicious cycle. Nowadays, few studies focus on the mutual and direct effects between physical and mental states with ocular surface states in autoimmune rheumatic patients [15].

Here, we selected five questionnaires to provide a comprehensive assessment of the physical and mental states of RA, SLE and SS patients. The Ocular Surface Disease Index (OSDI) is the most widely used questionnaire to assess subjective ocular surface conditions in DED patients, including the frequency of ocular symptoms [16, 17]. The Short Form 36-Health Survey (SF-36) is a general quality of life questionnaire developed by the American Medical Research Group [18, 19] and is wildly used, including the Physical Component Summary (PCS) and Mental Component Summary (MCS) subscales. The Health Assessment Questionnaire-Disability Index (HAQ-DI) measures difficulties in activities of daily living [20]. It’s originally developed to assess physical function in autoimmune rheumatic patients [21]. The Hospital Anxiety and Depression Scale (HADS) is one of the most commonly used tools for screening anxiety and depression in chronic physical diseases [22–24]. The Pittsburgh Sleep Quality Index (PSQI) is a 19-item self-report questionnaire measuring subjective sleep quality in patients with organic or nonorganic sleep disorders over the past 1 month [25, 26].

Therefore, in addition to collect ocular surface signs and symptoms, we also used several scales to evaluate the physical and emotional states (sleep, mood, quality of life, mental state, etc.) of patients during hospitalization to explore whether the various conditions are directly related to the ocular surface disorders, aiming at better understanding and treatment of ocular surface disorders in patients with autoimmune rheumatic diseases.

## Methods

### Research objects

Patients between 18–80 years old diagnosed with RA, SLE or primary SS (pSS) who were hospitalized in the Department of Rheumatology, The Second Xiangya Hospital, Central South University between March 2021 and July 2022 were randomly included in the study in equal proportion. The diagnostic criteria of RA, SLE and SS were based on the 2010 American College of Rheumatology (ACR)/European League Against Rheumatism (EULAR) classification criteria for RA [27, 28], 2019 EULAR/ACR Classification Criteria for SLE [29, 30] and 2016 EULAR/ACR Classification Criteria for SS [31] respectively. The control group consisted of volunteers without autoimmune rheumatic diseases matched for sex and age. The inclusion and exclusion criteria are as follows.

- Disease group inclusion criteria: a) age: 18–80, b) diagnosed with RA, SLE and pSS by rheumatologist.
- Control group inclusion criteria: a) age: 18–80, b) no autoimmune rheumatic diseases.
- Exclusion criteria: a) history of eye surgery, b) history of eye trauma, c) ocular medication history within 1 month, d) lactating or pregnant women, e) computer workers, f) diabetes, g) hypertension, h) cardiovascular disease, i) thyroid-associated ophthalmopathy, j) eyelid eversion, k) contact lens within 1 month.

All subjects participated in the program voluntarily and signed informed consent forms. This study received approval from the clinical Research Ethics Committee of The Second Xiangya Hospital of Central South University (Ethics No.: Ocular Surface Observation in patients with systemic immune Diseases, LYF2021028).

### Assessment for ocular surface

#### Subjective assessment

The OSDI is a 12-item questionnaire that evaluates ocular symptoms, environmental factors, and restricted activities over the past week. The total OSDI score is calculated as follows: OSDI score = (Sum of scores for all questions answered × 100) / (Numbers of questions answered × 4).

The total score ranges from 0 to 100, with higher scores indicating more serious subjective symptoms of ocular surface disease [32]. To facilitate evaluation, we classified the OSDI into 4 levels: 0 (0–12), 1 (13–22), 2 (23–32), 3 (≥ 33). Level 1, 2 and 3 were regarded as abnormal and also indicated the severity of dry eye [9, 16].

#### Objective assessment

##### Schirmer I test

Reflective tear secretion levels were measured by the Schirmer I test. A tear secretion test paper was placed in 1/3 of the temporal conjunctival sac of the lower eyelid, and the patient was instructed to close his or her eyes gently for 5 min. The wetted length of the tear secretion test paper was recorded. Tear secretion ≤ 10 mm/5 min was considered abnormal [16, 33].

##### TBUT

The tear break-up time (TBUT) was observed by fluorescein sodium staining, detecting the stability of the tear film. The patient was instructed to blink 3 times after 1–2 drops of sodium fluorescein dropped into the conjunctiva sac of the lower eyelid. After the last blink, the patient opened his or her eyes naturally. The time when the first black spot appeared on the cornea was observed under cobalt-blue light was the break-up time of the tear film. The average value was taken for 3 repeated measurements. A TBUT ≤ 10 s was considered abnormal [33].

##### CFS

The corneal fluorescein staining (CFS) was also observed under cobalt- blue light after fluorescein sodium staining and graded by the OXFORD scale [34]. Grade 0 was regarded as normal, and grades 1, 2, 3 and 4 were regarded as abnormal.

##### Meibomian gland secretion

Observation of the secretions of the meibomian gland: The thumb and index finger were used to turn the upper and lower eyelids and gently extrude, and the secretions of the upper and lower eyelids were observed and scored. Grade 0 indicated clear and transparent liquid secretions, representing normal secretion. Grade 1 indicated cloudy liquid, grade 2 indicated cloudy granular secretions and grade 3 indicated secretions as thick as toothpaste, reflecting abnormal secretion [35].

##### LIPCOF

Lid-parallel conjunctival folds (LIPCOF) were evaluated, without fluorescein, on the bulbar conjunctiva in the area perpendicular to the temporal and nasal limbus, above the lower lid. They were classified by counting the number of folds. 0, no conjunctival folds; 1, one permanent and clear parallel fold; 2, two permanent and clear parallel folds; 3, more than two permanent and clear parallel folds. A score of 0 was considered normal, and other scores (including 1, 2, 3) were considered abnormal [36].

##### Symblepharon and corneal clarity

Symblepharon presence and corneal clarity were observed under the slit lamp.

#### Diagnosis, classification and severity of dry eye

According to the TFOS DEWS II [16, 37–39], only the patients who had both symptoms and signs of DED were identified with DED. Symptoms were measured by OSDI, including photophobia, foreign body sensation, pain and blurred vision, restrictions on daily activities (reading, television and computer viewing, and driving at night), and influences of environmental factors on eyes (sandstorm weather or dry environment). A score of OSDI ≥ 13 was considered abnormal. Signs of dry eye included TBUT ≤ 10 s, CFS ≥ Grade 1. Considering the separation of signs and symptoms of dry eye, DED can also be diagnosed when patients had mild or no symptoms but were accompanied by severe damage of tear function and ocular surface. For sub-classification, when DED patients had abnormal Schirmer I test (≤ 10 mm/5 min) and a normal meibomian gland, were characterized as an aqueous deficiency type [40]. When DED patients had abnormal meibomian gland function with normal tear secretion, were characterized as evaporative dry eye, while the rest were characterized as mixed dry eye. The severity of dry eye was evaluated according to the instructions of DEWS I [37].

### Systemic questionnaires

The SF-36 provides specific scores in eight categories, as well as mental, physical component scores and composite score. The Physical Component Summary (PCS) includes physical function (PF), role physical (RP), body pain (BP), and general health (GH), while the Mental Component Summary (MCS) includes vitality (VT), social function (SF), role emotion (RE), and mental health (MH). The higher the score, the better the health-realted quality of life [41].

The 20-item HAQ-DI assesses 8 aspects, including eating, walking, dressing, arising, hygiene, reaching, griping, and common daily activities. It’s rated on a scale of 0 to 3 for each question, where 0 means “no difficulty” and 3 means “cannot do it,” with extra points if the specific activity requires auxiliary equipment or equipment. Therefore, a higher score indicates poorer function. The HAQ-DI score is a continuous variable and was transformed into a categoric variable to represent six different health states ([0–0.5], [0.5–1.0], [1.0–1.5], [1.5–2.0], [2.0–2.5] and [2.5–3.0]) [20, 42, 43].

The HADS scale, consisting of 14 items, which are divided into two subscales, provides an assessment of both anxiety and depression and grades patients into three levels based on their scores. A score of 0–7 indicates no symptoms, 8–10 indicates a tendency to anxiety or depression, and 11–21 indicates anxiety or depression [23].

The PSQI includes 19 individual items, which were aggregated into 7 components that assessed various aspects of sleep, including subjective sleep quality, time to sleep, sleep duration, sleep efficiency, sleep disorder, use of hypnotic drugs and daytime dysfunction. The sum of these 7 components produced a total score that was used to distinguish between "good" and "poor" sleepers. A higher PSQI score indicates poorer sleep quality [25, 26].

### Statistics

Descriptive statistics were used to assess the characteristics of the subjects. Comparisons of continuous variables were performed using the independent samples t- test. The chi-square test was used to compare categorical variables, while the rank variables were tested by the Wilcoxon rank sum test. Pearson and spearman’s correlation analysis were used to assess the association between systemic questionnaires and ocular surface disorders. *P* < 0.05 was regarded as statistically significant. Samples with incomplete data were excluded.

## Results

### Characteristics of participants

A total of 240 eyes of 120 participants (180 eyes of 90 patients and 60 eyes of 30 controls) were included in this study. The disease group consisted of 30 rheumatoid arthritis, 30 systemic lupus erythematosus and 30 primary Sjogren's syndrome patients, including 77 females and 13 males, with an average age of 49.87 ± 14.89 years (range 18–80). The control group included 30 volunteers (24 females and 6 males) with an average age of 43.13 ± 20.29 years (range 18–78). The differences in age and sex between these two groups were not statistically significant (Table 1).

**Table 1:** The clinical information and ocular surface disorders of the study participants

Characteristics	Autoimmune rheumatic patients	Controls	*P* Value
**Subjects (n)**	90	30	
**Age (years)**	49.87 ± 14.89	43.13 ± 20.29	0.10
**Gender-Female, n (%)**	77 (85.56)	24 (80.00)	0.47
Gender ratio-Female/Male (age 18–40), n	26: 1	12: 2	0.27
Gender ratio-Female/Male (age 41–60), n	34: 7	4: 2	0.32
Gender ratio-Female/Male (age 61–80), n	17: 5	8: 2	0.86
**OSDI score**	17.92 ± 17.40	10.07 ± 14.75	0.03*
**OSDI level, n (%)**			0.02*
0 (0–12)	46 (51.11)	24 (80.00)	
1 (13–22)	17 (18.89)	3 (10.00)	
2 (23–32)	8 (8.89)	0	
3 (≥ 33)	19 (21.11)	3 (10.00)	
**Eyes (n)**	180	60	
**Schirmer I test (mm/5 min)**	9.19 ± 8.44	12.80 ± 10.25	0.007*
**Schirmer I test level, n (%)**			0.15
Normal (> 10 mm/5 min)	65 (36.11)	28 (46.67)	
Abnormal (≤ 10 mm/5 min)	115 (63.89)	32 (53.33)	
**TBUT (s)**	5.76 ± 3.19	6.40 ± 3.41	0.18
**TBUT level, n (%)**			0.73
Normal (> 10 s)	21 (11.67)	8 (13.33)	
Abnormal (≤ 10 s)	159 (88.33)	52 (86.67)	
**Corneal fluorescein staining, n (%)**			< 0.001*
Normal (Grade 0)	134 (74.44)	57 (95.00)	
Abnormal (≥ Grade 1)	46 (25.56)	3 (5.00)	
**Meibomian gland secretion, n (%)**			0.23
Normal (Grade 0)	106 (58.89)	30 (50.00)	
Abnormal (≥ Grade 1)	74 (41.11)	30 (50.00)	
**LIPCOF, n (%)**			0.003*
Normal (Grade 0)	135 (75.00)	49 (81.67)	
Abnormal (≥ Grade 1)	45 (25.00)	11 (18.33)	
**Symblepharon presence, n (%)**			-
Normal	180 (100)	60 (100)	
Abnormal	0	0	
**Corneal clarity, n (%)**			0.56
Normal	178 (98.89)	60 (100)	
Abnormal	2 (1.11)	0	
**Dry eye level, n (%)**			< 0.001*
No dry eye	86 (47.78)	47 (78.33)	
I	31 (17.22)	2 (3.33)	
II	36 (20.00)	6 (10.00)	
III	27 (15.00)	5 (8.33)	
IV	0	0	
**Classification of dry eye, n (%)**			< 0.001*
No dry eye	86 (47.78)	47 (78.33)	
Aqueous deficiency dry eye	37 (20.56)	6 (10.00)	
Evaporative dry eye	29 (16.11)	2 (3.33)	
Mixed dry eye	28 (15.56)	5 (8.33)	

*OSDI* Ocular Surface Disease Index, *TBUT* Tear film break-up time, *LIPCOF* Lid-parallel conjunctival folds, *n* number, *mm* millimeter, *min* minute, *s* second

*P* value means autoimmune rheumatic patients compared to controls, *P < 0.05

### Ocular surface characteristics of the participants

As shown in Table 1, autoimmune rheumatic patients had more severe ocular surface symptoms than controls measured by the OSDI score (17.92 ± 17.40 and 10.07 ± 14.75, respectively, *P* = 0.03). Among them, 48.89% of patients (44 in 90) had abnormal OSDI score, compared with 20.00% of controls (6 in 30). A significant difference was also found in the OSDI level between the two groups (*P* = 0.02).

For evaluation of ocular surface signs, autoimmune rheumatic patients showed less tear secretion than controls measured by Schirmer I test (9.19 ± 8.44 and 12.80 ± 10.25, respectively, *P* = 0.007). Schirmer I test was ≤ 10 mm/5 min in 115 (63.89%) patients, compared with 32 (53.33%) controls. No statistically significant difference was detected in Schirmer I test level between the two groups (Table 1, *P* = 0.15). 88.33% of eyes (159 in 180) of patients and 86.67% of eyes (52 in 60) of controls had abnormal TBUT (Table 1, *P* = 0.73). For corneal fluorescein staining, 25.56% of eyes (46 in 180) of patients had abnormal CFS grade and significantly higher than 5.00% of eyes (3 in 60) of controls (*P* < 0.001). Interestingly, there were more abnormal meibomian gland secretions in the control group than in the patients (50.00% and 41.11%, respectively) but the difference was not statistically significant (*P* = 0.23). The LIPCOF grade was abnormal in 25.00% of autoimmune rheumatic patients and 18.33% of controls (Table 1, *P* = 0.003). Additionaly, no eyes were found to have symblepharon and only 1.11% of eyes (2 in 180) of patients had abnormal corneal clarity. No statistically significant difference was detected in corneal clarity between the two groups (Table 1, *P* = 0.56).

In general, 94 in 180 (52.22%) eyes of autoimmune rheumatic patients were diagnosed with dry eye, while the incidence shown 21.67% (13 in 60 eyes) in controls (Table 1, *P* < 0.001). For the severity of dry eye, most rheumatic immune patients and controls were at level II (20.00% and 10.00%, respectively). For autoimmune rheumatic patients, aqueous deficiency dry eye accounted for the majority (20.56%), while evaporative and mixed dry eye were less (16.11% and 15.56%, respectively). For the control group, aqueous deficiency dry eye was the most common type (10.00%), followed by mixed and evaporative type (8.33% and 3.33%, respectively). The differences were statistically significant in dry eye level and classification between the two groups (Table 1, *P* < 0.001).

### Physical and mental scales in participants

#### SF-36 scale

**Fig. 1:**
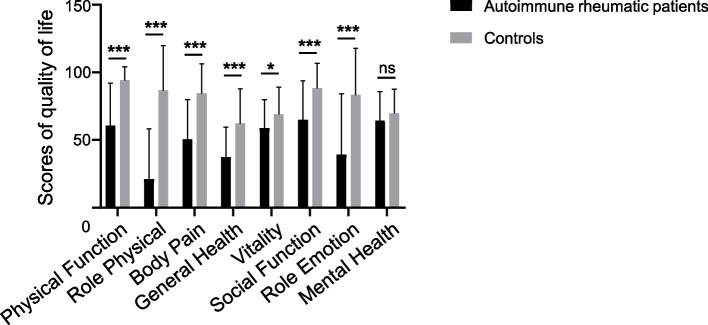
Comparison of SF-36 subscales between autoimmune rheumatic patients and controls. *, *P* < 0.05; ***, *P* < 0.001; ns, not significant. Bars represent the means; error bars represent the standard error of the mean

The SF-36 has been wildly used to assess health-related quality of life in patients with autoimmune rheumatic diseases. A higher score indicates a better quality of life. As shown in Table 2, lower SF-36 composite scores were shown in autoimmune rheumatic patients than in controls, indicating that autoimmune rheumatic diseases obviously reduced patients’ health-related quality of life (*P* < 0.001). For the sub-indicators PCS and MCS, autoimmune rheumatic patients also had lower scores, indicating poorer physical and mental quality (*P* < 0.001). Among SF-36 subscales, the physical states (including PF, RF, BP and GH categories) and mental states (including VT, SF and RE categories) were also significantly worse in autoimmune rheumatic patients than that in control group (Table 2, Fig. 1, *P* < 0.001). Among these, RP was the strongest indicator between patients and controls. The difference in MH (representing mental state) between the two groups was not statistically significant (Table 2, Fig. 1, *P* = 0.26).

**Table 2:** SF-36 characteristics of the study participants

	**Autoimmune rheumatic patients (*n* = 90)**	**Controls (*n* = 30)**	***P* Value**
**Composite score**	49.65 ± 20.43	79.66 ± 18.19	< 0.001*
PCS score	42.34 ± 22.55	81.78 ± 19.11	< 0.001*
MCS score	56.99 ± 23.73	77.55 ± 19.26	< 0.001*
PF score	60.56 ± 31.52	94.04 ± 10.10	< 0.001*
RP score	21.06 ± 37.08	86.54 ± 33.34	< 0.001*
BP score	50.31 ± 29.45	84.42 ± 21.92	< 0.001*
GH score	37.44 ± 22.21	62.12 ± 25.85	< 0.001*
VT score	58.72 ± 21.10	68.85 ± 20.12	0.03*
SF score	64.86 ± 28.72	88.46 ± 18.34	< 0.001*
RE score	39.00 ± 45.23	83.33 ± 34.32	< 0.001*
MH score	64.36 ± 21.34	69.54 ± 18.10	0.26

*SF-36* Short Form 36-Health Survey, *PCS* Physical Component Summary, *MCS* Mental Component Summary, *PF* Physical Function, *RP* Role Physical, *BP* Body Pain, *GH* General Hhealth, *VT* Vitality, *SF* Social Function, *RE* Role Emotion, *MH* Mental Health, *n* number

*P* value means autoimmune rheumatic patients compared to controls, *P < 0.05

#### HAQ-DI scale

The HAQ-DI was used to assess difficulties in activities of daily living. The autoimmune rheumatic patients had more difficulties than the control group (Table 3, 0.64 ± 0.85 and 0.04 ± 0.3, respectively, *P* < 0.001). All control participants were at level 1, while 66.67% of patients (60 in 180) were at level 1 and the rest were worse (Table 3, *P* = 0.01).

**Table 3:** HAQ-DI characteristics of the study participants

	**Autoimmune rheumatic patients (*n* = 90)**	**Controls (*n* = 30)**	***P* value**
**HAQ-DI score**	0.64 ± 0.85	0.04 ± 0.3	< 0.001*
**HAQ-DI level, n (%)**			0.001*
1	60 (66.67)	30 (100)	
2	6 (6.67)	0	
3	8 (8.89)	0	
4	5 (5.55)	0	
5	8 (8.89)	0	
6	3 (3.33)	0	

*HAQ-DI* Health Assessment Questionnaire-Disability Index, *n* number

*P* value means autoimmune rheumatic patients compared to controls, *P < 0.05

#### HADS scale

The HADS assesses anxiety and depression. The anxiety scores were significantly higher in autoimmune rheumatic patients than controls (6.89 ± 4.37 and 4.92 ± 3.78, respectively, *P* = 0.04). And the depression scores were 7.53 ± 4.54 and 6.54 ± 4.38 in participants with or without autoimmune rheumatic diseases, respectively (*P* = 0.32).

#### PSQI scale

The PSQI was used to assess sleep quality. The sleep quality of autoimmune rheumatic patients was poorer than controls (8.84 ± 4.86 and 7.00 ± 3.91, respectively, *P* = 0.07).

### Associations between ocular surface disorders and psychological, physiological states

**Fig. 2:**
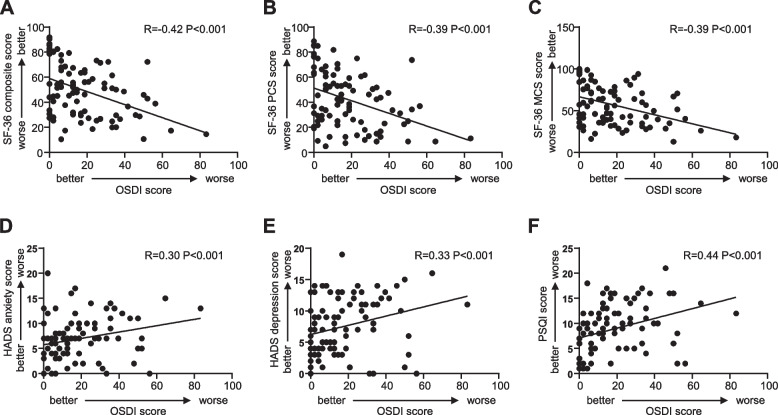
Associations between OSDI score and psychological, physiological states in autoimmune rheumatic patients. **A** Association between OSDI score and Short Form 36-Health Survey (SF-36) composite score. R (Pearson correlation coefficient): -0.42, *P* < 0.001. **B** Association between OSDI score and SF-36 physical component summary (PCS). R: -0.39, *P* < 0.001. **C** Association between OSDI score and SF-36 mental component summary (MCS). R: -0.39, *P* < 0.001. **D** Association between OSDI score and Hospital Anxiety and Depression Scale (HADS) anxiety subscale. R: 0.30, *P* < 0.001. **E** Association between OSDI score and HADS depression subscale. R: 0.33, *P* < 0.001. **F** Association between OSDI score and Pittsburgh Sleep Quality Index (PSQI) score. R: 0.44, *P* < 0.001

We analyzed the correlation between ocular surface disorders and questionnaire indicators, and found that in autoimmune rheumatic patients, the OSDI score showed a statistically significant correlation with the SF-36 composite score, as well as all the SF-36 indicators (Table 4, Fig. 2A-C, SF-36: r = -0.42, *P* < 0.001, PCS: r = -0.39, *P* < 0.001, MCS: r = -0.39, *P* < 0.001), showing a significant association between increased ocular surface symptoms and decreased quality of life. In addition, the OSDI score was also significantly associated with anxiety, depression and PSQI score (Table 4, Fig. 2D-F, anxiety: r = 0.30, *P* < 0.001, depression: r = 0.33, *P* < 0.001, PSQI: r = 0.44, *P* < 0.001). However, there was no significant correlation between HAQ-DI and the OSDI.

**Table 4:** Associations between ocular surface disorders and psychological, physiological states in autoimmune rheumatic patients

Factors	OSDI	Schirmer I	TBUT	CFS	MGS	LIPCOF	DEL
**SF-36**							
Composite score	-0.42**	0.05	-0.01	0.19	-0.10	0.09	-0.15
PCS score	-0.39**	0.10	-0.09	0.18	-0.05	0.10	-0.14
MCS score	-0.39**	0.01	0.08	0.17	-0.14	0.07	-0.14
PF score	-0.30**	0.11	-0.06	0.17	0.02	0.09	-0.14
RP score	-0.34**	0.08	-0.16	0.19	0.03	-0.04	-0.02
BP socre	-0.24**	0.11	0.01	0.11	-0.03	0.17	-0.13
GH score	-0.36**	-0.04	-0.02	0.19	-0.19	0.06	-0.14
VT score	-0.34**	0.03	0.06	0.15	-0.19	0.07	-0.15
SF score	-0.32**	-0.12	0.08	0.16	-0.13	0.03	-0.12
RE score	-0.28**	0.06	0.08	0.16	-0.07	0.09	-0.05
MH score	-0.35**	0.08	0.02	0.07	-0.06	0.03	-0.18
**HADS**							
Anxiety	0.30**	-0.06	0.08	-0.02	-0.05	-0.13	0.16
Depression	0.33**	-0.06	0.05	-0.02	-0.05	0.02	0.18
**PSQI**	0.44**	-0.12	-0.14	-0.03	0.14	0.08	0.20
**HAQ-DI**	0.18	-0.10	-0.02	-0.13	0.06	-0.07	0.09

*OSDI* Ocular Surface Disease Index, *TBUT* Tear film break-up time, *CFS* Corneal fluorescein staining, *MGS* Meibomian gland secretion, *LIPCOF* Lid-parallel conjunctival folds, *DEL* Dry eye level, *SF-36* Short Form 36-Health Survey, *PCS* Physical Component Summary, *MCS* Mental Component Summary, *PF* Physical Function, *RP* Role Physical, *BP* Body Pain, *GH* General Health, *VT* Vitality, *SF* Social Function, *RE* Role Emotion, *MH* Mental Health, *HADS* Hospital Anxiety and Depression Scale, *PSQI* Pittsburgh Sleep Quality Index, *HAQ-DI* Health Assessment Questionnaire-Disability Index

*P* value means dry eye disease compared to systemic questionnaires among autoimmune rheumatic patients, **P < 0.01

We further analyzed the correlation of ocular surface signs, dry eye level and psychological, physiological states. There were no statistically significant correlations detected in autoimmune rheumatic patients between questionnaire indicators and Schirmer I, TBUT, CFS, meibomian gland secretion, LIPCOF, dry eye level (Table 4).

However, in control group, the OSDI score was correlated with the SF-36 subscale PF and RE score (Supplementary Table S1, PF: r = -0.33, *P* = 0.02, RE: r = -0.35, *P* = 0.01), and had no statistically significant correlations with other SF-36 indicators. In the same way, the OSDI score was also significantly associated with PSQI score (Supplementary Table S1, r = 0.49, *P* < 0.001). However, there was no significant correlation between anxiety, depression, HAQ-DI and the OSDI. In addition, the Schirmer I showed statistically significant correlations with SF-36 subscale PCS score, PF score, GH score, SF score (Supplementary Table S1, PCS: *r* = 0.41, *P* = 0.04, PF: *r* = 0.44, *P* = 0.02, GH: *r* = 0.45, *P* = 0.02, SF: *r* = 0.44, *P* = 0.03). There were no statistically significant correlations detected in control group between questionnaire indicators and TBUT, CFS, meibomian gland secretion, LIPCOF, dry eye level (Supplementary Table S1).

## Discussion

Autoimmune rheumatic diseases are chronic conditions characterized by an overactive and uncontrolled immune response, involving multiple organs of the body (including eyes) [1, 3, 44, 45]. At present, the multifactorial pathogenesis is still partially understood [46]. As the disease has a long course and is difficult to cure, patients often suffer from both physical pain and mental stress. Additionally, patients with autoimmune rheumatic diseases also have an increased burden of life [1, 3, 45, 47].

Our study demonstrated that autoimmune rheumatic patients had poor health-related quality of life compared to controls. Both physical and mental health quality were reduced. Among these, role physical (role limitations due to physical health problems) was the most affected indicator. Our finding is in accord with previous reports [48, 49], and perhaps suggests the potential benefit of the patient's physical and mental health care as part of a comprehensive management of autoimmune rheumatic diseases [48, 50]. Moreover, autoimmune rheumatic diseases exacerbate the difficulty of daily living. This is consistent with Lorand's study [51]. Autoimmune rheumatic patients are often unable to take care of themselves due to the involvement of vital organs such as joints, lungs and kidneys [1, 2]. Studies have shown that autoimmune rheumatic diseases can increase levels of depression and anxiety and decrease sleep quality [52–55]. Our study also revealed that autoimmune rheumatic patients were more anxious than controls. However, depression and sleep quality had no differences between the two groups. At present, in addition to conventional disease-modifying antirheumatic drugs (DMARDs) and glucocorticoid-sparing regimens, psychological interventions (for example, relaxation, stress management and cognitive coping skills) are recommended to help these patients adjust to living with their condition and have the potential to increase their quality of life and life expectancy. National Institute for Health and Care Excellence (NICE) guidelines (https://www.nice.org.uk/guidance/ng100/chapter/Recommendations) stipulate that these patients should be periodically assessed for the impact of their disease on their lives, including health-related quality of life and mood.

Patients with autoimmune rheumatic diseases often have ocular manifestations, especially ocular surface disorders such as conjunctivitis, keratitis and dry eye disease [56]. An observational study has shown that 46% meibomian gland dysfunction, 49% superficial punctate keratopathy and 44% shortened TBUT are observed in patients with primary and secondary Sjögren syndrome [57]. Our study indicated that among autoimmune rheumatic patients, 48.89% ocular symptoms, 63.89% reduced tear secretion, 88.33% shortened TBUT, 25.56% corneal fluorescein staining, 41.11% abnormal meibomian gland secretion, 25% LIPCOF, no symblepharon and 1.11% corneal opacity are showed. In addition, 52.22% of eyes of patients are diagnosed with DED with 20.56% aqueous deficiency type, 16.11% evaporative type and 15.56% mixed type. Autoimmune rheumatic diseases are associated with an overactive and uncontrolled immune response, the tear glands of autoimmune rheumatic patients attacked by autoantibodies often fail to secrete tears [58]. In our study, patients produced fewer tears than the control group. This observation is in accordance with Guannan’s and Kim’s [59] study. We futher found that the difference in tear film stability was not statistically significant between patients with autoimmune rheumatic diseases and controls. This result is also consistent with the study of Usuba [60] and Marsovszky [61]. The mechanism of DED in most autoimmune rheumatic diseases is focal lymphocytic infiltration and chronic inflammation of the exocrine glands rather than meibomian gland dysfunction [58, 62–64]. This is the reason why most types of DED associated with autoimmune rheumatic diseases are the aqueous-deficient type [38, 65, 66]. Corneal fluorescein staining is an important index to evaluate the severity of dry eye [37]. Autoimmune rheumatic patients had more severe corneal staining than controls. Liang’s research also confirms this trend [67]. From what has been discussed above, ocular involvement in autoimmune rheumatic patients is more severe than that in people without autoimmune rheumatic diseases.

Currently, there is a growing belief that ocular surface disorders especially DED are associated with psychological, psychosocial and social factors, especially anxiety and depression [68, 69]. Studies have shown that in a healthy population, reduced sleep duration, higher psychological stress, and poorer self-perceived health status were independently associated with DED [70]. Therefore, we should not only focus on the pathological damage of autoimmune rheumatic disease on ocular surface but also pay attention to the indirect impact of the psychological problems caused by the disease. Due to the higher incidence of ocular surface disorders in Sjogren's syndrome (SS), relevant research has focused on SS at present. Studies have demonstrated that autoimmune rheumatic patients have a reduced quality of life, poorer sleep quality and an increased risk of anxiety and depression [49, 71–73]. For patients with SS, psychological factors significantly impact the quality of life, wellbeing, cognitive functions and disease activity of the patients [74, 75]. Additionally, ocular symptoms including pain, dryness, and itching, pose serious physical limitations and cause psychological distress [76]. Researchers suggest that the biopsychosocial perspective is crucial for treatment [77]. However, there is a lack of research on the correlation between the physical and mental state and ocular surface disorders in patients with rheumatoid arthritis (RA) and systemic lupus erythematosus (SLE).

Our study demonstrate that unlike controls, lower health-related quality of life, more anxiety and depression, poorer sleep quality were moderately associated with DED symptoms among autoimmune rheumatic patients. DED is a disease in which symptoms and signs are separated according to DEWS II[16]. No signficant association was observed between other ocular surface signs and systemic factors. It indicates that DED affects health-related quality of life, anxiety, depression and sleep quality of rheumatic immune patients more than people without autoimmune rheumatic diseases. These systemic conditions can aggravate DED [70]. Management of systemic problems is essential when treating autoimmune rheumatic patients. Improving the patient's physical and mental condition is also beneficial to DED. As a series of systematic chronic diseases, autoimmune rheumatic diseases are expected to pay more attention to the patients' physical and mental state while focusing on ocular involvement [78]. Holistic thinking is necessary when treating a patient with autoimmune rheumatic disease-related ocular involvement. It is important to address comorbid conditions such as depression and poor sleep quality. These conditions are thought to exacerbate one another and contribute to the overall burden of disease [10, 79]. In addition, patients with dry eye disease sometimes show the different disease state of the two eyes. Perhaps the same patient in a state of anxiety is diagnosed with dry eye in one eye while the other eye is normal. In order to avoid potential bias, we used of averaged ocular surface indexes of both eyes of the same patient to conduct the correlation analysis with the systemic indicators [80].

In addition, the influence of gender and age on DED cannot be ignored, although in our study age and sex are matched. The risk of DED increases with age in both sexes, while its incidence is higher among females. In addition, the condition of menopause in aging women may also contribute to dry eye onset or worsening as a consequence of an overall hormonal imbalance [81–83]. Autoimmune rheumatic diseases are also more common in women. For different age stages of autoimmune rheumatic patients, the ocular surface conditions are different. We need to pay more attention to the changes in hormone levels to observe whether it is hormone that causes the severity of dry eye, rather than the primary disease. This also needs more research in the future.

There are some limitations in this study. First, the disease group we studied only included hospitalized patients and ignored non-hospitalized patients. Hospitalized patients with autoimmune rheumatism are more severe and have more comorbidities than those who are not hospitalized. This will cause bias to some extent. Second is the small sample size. Finally, due to the non-representative nature of participants, the volunteer bias can’t be ignored.

In general, autoimmune rheumatic patients have poorer health-related quality of life, more difficulties in daily living and suffer from ocular surface disorders. Factors including lower quality of life, more anxiety and depression, and poorer sleep quality are associated with DED symptoms in autoimmune rheumatic patients. Management of systemic conditions and psychotherapy should also be considered as part of the treatment and have benefits for DED among autoimmune rheumatic patients.

## Supplementary Information

**Additional file 1:** **Supplementary Table S1.** Associations between ocular surface disorders and psychological, physiological states in control group.

## Data Availability

The data that support the findings of this study are available from the corresponding author upon reasonable request.

## Abbreviations

BP: Body pain
CFS: Corneal fluorescein staining
DED: Dry eye disease
GH: General health
HADS: Hospital Anxiety and Depression Scale
HAQ-DI: Health Assessment Questionnaire-Disability Index
LIPCOF: Lid-parallel conjunctival folds
MCS: Mental Component Summary
MH: Mental health
OSDI: Ocular Surface Disease Index
PCS: Physical Component Summary
PF: Physical function
PSQI: Pittsburgh Sleep Quality Index
RP: Role physical
pSS: Primary Sjogren's syndrome
RA: Rheumatoid arthritis
RE: Role emotion
SF: Social function
SF-36: Short Form 36-Health Survey
SS: Sjogren's syndrome
SLE: Systemic lupus erythematosus
TBUT: Tear break-up time
VT: Vitality
