# Supplementary material for: Evaluation and correlation analysis of ocular surface disorders and quality of life in autoimmune rheumatic diseases: a cross-sectional study
Source: BMC Ophthalmol. 2023 May 22;23:229. doi: 10.1186/s12886-023-02959-5 (PMC10201733; doi:10.1186/s12886-023-02959-5)
Supplement: Supplementary file 1 — Additional file 1: Supplementary Table S1. Associations between ocular surface disorders and psychological, physiological states in control group. [file 12886_2023_2959_MOESM1_ESM.docx]

**Supplementary Table S1.** Associations between ocular surface disorders and psychological, physiological states in control group

| **Factors** | **OSDI** | **Schirmer I** | **TBUT** | **CFS** | **MGS** | **LIPCOF** | **DEL** |
| --- | --- | --- | --- | --- | --- | --- | --- |
| **SF-36** |  |  |  |  |  |  |  |
| Composite score | -0.19 | 0.27 | 0.20 | 0.02 | -0.09 | -0.18 | -0.29 |
| PCS score | -0.20 | 0.41* | 0.26 | -0.03 | -0.03 | -0.19 | -0.27 |
| MCS score | -0.12 | 0.27 | 0.12 | 0.06 | -0.14 | -0.16 | -0.09 |
| PF score | -0.33* | 0.44* | 0.24 | -0.05 | 0.06 | -0.14 | -0.29 |
| RP score | -0.23 | 0.31 | 0.25 | 0.01 | -0.18 | -0.24 | -0.31 |
| BP socre | -0.01 | 0.24 | 0.14 | 0.09 | 0.15 | 0.04 | -0.26 |
| GH score | -0.21 | 0.45* | 0.23 | -0.16 | -0.01 | -0.23 | -0.23 |
| VT score | -0.07 | 0.16 | 0.14 | -0.08 | 0.10 | -0.14 | -0.10 |
| SF score | -0.19 | 0.44* | 0.16 | 0.16 | -0.14 | -0.11 | 0.03 |
| RE score | -0.35* | 0.28 | 0.14 | 0.08 | -0.22 | -0.20 | -0.20 |
| MH score | -0.03 | 0.07 | -0.05 | 0.03 | -0.15 | -0.02 | 0.08 |
| **HADS** |  |  |  |  |  |  |  |
| Anxiety | -0.01 | -0.25 | -0.13 | 0.09 | 0.12 | 0.08 | 0.04 |
| Depression | 0.03 | -0.05 | -0.12 | 0.20 | -0.03 | 0.13 | 0.24 |
| **PSQI** | 0.49** | -0.22 | -0.25 | 0.02 | -0.13 | 0.28 | 0.21 |
| **HAQ-DI** | 0.07 | -0.23 | -0.10 | -0.09 | -0.08 | -0.13 | 0.31 |

OSDI = Ocular Surface Disease Index, TBUT = Tear film break-up time, CFS = Corneal fluorescein staining , MGS = Meibomian gland secretion, LIPCOF = Lid-parallel conjunctival folds, DEL = Dry eye level, SF-36 = Short Form 36-Health Survey, PCS = Physical Component Summary, MCS = Mental Component Summary, PF = Physical Function, RP = Role Physical, BP = Body Pain, GH = General Health, VT = Vitality, SF = Social Function, RE = Role Emotion, MH = Mental Health, HADS = Hospital Anxiety and Depression Scale, PSQI = Pittsburgh Sleep Quality Index, HAQ-DI = Health Assessment Questionnaire-Disability Index.

*P* value means dry eye disease compared to systemic questionnaires among autoimmune rheumatic patients, **P* < 0.05, ***P* < 0.01.
